# Radical Cure of Alveolar Abscess by Injection of Gutta Percha Solution

**Published:** 1887-02

**Authors:** D. R. Jennings


### Radical Cure of Alveolar Abscess by Injection of Gutta Percha Solution.

BY D. R. JENNINGS, D.D.S.

(Read before the Cleveland Dental Society.)

Alveolar abscess in the quite recent past and at the present time is pressing its claims upon the attention of the Dental profession, and like every other truth which has a practical bearing, and tends to the alleviation, elevation, and purification of humanity, must in its apprehension and reception pass through its incipiency and development to maturity; it will in its different stages of advancement meet with quick, generous, or unfriendly opposition. As has been remarked by some one, alveolar abscess has no written history. The pathology of alveolar abscess is characterized by inflammation presenting distinct stages, irritative, congestive, and exudating. The first is purely that of an irritant; the second is characterized by intense redness, increased heat with pain and local swelling; this is followed by the third and last stage, the exudating or pus discharging—*i. e.* suppuration. Now it has arrived at the first real stage of alveolar abscess, and I think, demands an entirely different and distinct treatment from any that I have seen written or taught by any of our practitioners. In this matter I want to thoroughly impress upon the profession that this treatment is for the curing of alveolar abscess, not for simply congested, or sore teeth, or inflamed periosteum; although I think it the best treatment in such cases I have ever had any thing to do with.

You must always remember that in this treatment, like all others, to arrive at success you must be very thorough, leaving nothing to *luck*. You will remember also, that there can be no alveolar abscess unless there is absorbtion of the alveolus, and that this absorbtion makes a cavity, and that cavity must be disposed of to effect a cure. My plan of procedure in such cases is: As soon as there is an abscess formed you will find, if you extract the tooth, that the root of the tooth has become denuded of its periosteum where the sac is attached. The objective point is to get rid of the abscess and restore to a healthy condition.

After trying all the remedies recommended by others and having failures in quite a large per cent. of cases, I tried the plan of filling the whole of the abscess cavity and root canal with a solution of gutta percha in chloroform. To make this, take a portion of base plate gutta percha; cut it into small pieces and put into a bottle containing chloroform, enough to make a paste of the consistency of thin cream. Clean the pulp chamber, root canal, and abscess cavity thoroughly--exhausting all the pus from the sack at and around the roots—wash with alcohol and water equal parts, or with per oxide of hydrogen; dry as well as you can. Then with one of Donaldson's little bristles, made for cleansing root canals, with cotton fibers wrapped around it, dip into the gutta percha solution and introduce into the pulp chamber and root or roots as the case may be, using the cotton wrapped broach as a piston to pump the solution through the root canal into the cavity of the abscess, continuing to force the solution through the root until it makes its appearance at the sinus opening. If it is found coming through too freely, lay the finger on the opening, thus causing the solution to be forced into any and every place around the root where the sac is, in this manner strangulating it and preventing the gathering of lymph to be subsequently decomposed into pus. The abscess is thus destroyed. The gutta percha being an inert substance, becomes encysted, nature thus assisted goes on and closes up the sinus; and you will have no more fear than if there had never been an abscess. It has one more recommendation, to the patient at least; it is painless. I have pursued this course of treatment since 1879, and as far as I know, have not had a failure. I do not say that there has not been one; for you all very well know that they will not always come home to roost however much we may wish them to. Hoping that when you find an abscess that will not yield to any other treatment you will give this a fair trial, I shall have accomplished my purpose in presenting this plan for your consideration.

